# Differences and relationships between weightbearing and non-weightbearing dorsiflexion range of motion in foot and ankle injuries

**DOI:** 10.1186/s13018-024-04599-x

**Published:** 2024-02-03

**Authors:** Yuta Koshino, Tomoya Takabayashi, Hiroshi Akuzawa, Takeshi Mizota, Shun Numasawa, Takumi Kobayashi, Shintarou Kudo, Yoshiki Hikita, Naoki Akiyoshi, Mutsuaki Edama

**Affiliations:** 1 Faculty of Health Sciences, Hokkaido University, Kita 12, Nishi 5, Kita-Ku, Sapporo, 060-0812 Japan; 2 Institute for Human Movement and Medical Sciences, Niigata University of Health and Welfare, Niigata, Japan; 3 Department of Rehabilitation, Soejima Orthopedic Hospital, Takeo, Saga Japan; 4 Department of Rehabilitation, Takarazuka University of Medical and Health Care, Takarazuka, Japan; 5 Faculty of Health Science, Hokkaido Chitose College of Rehabilitation, Chitose, Japan; 6 Inclusive Medical Sciences Research Institute, Morinomiya University of Medical Sciences, Osaka, Japan; 7 Graduate School of Health Sciences, Morinomiya University of Medical Sciences, Osaka, Japan; 8 AR-Ex Medical Research Center, Tokyo, Japan; 9 Aruck Lab, Osaka, Japan; 10 Department of Rehabilitation, J Medical Oyumino, Chiba, Japan

**Keywords:** Ankle injury, Foot injury, Range of motion, Flexibility, Stiffness

## Abstract

**Background:**

This study aimed to: (1) identify assessment methods that can detect greater ankle dorsiflexion range of motion (DROM) limitation in the injured limb; (2) determine whether differences in weightbearing measurements exist even in the absence of DROM limitations in the injured limb according to non-weightbearing measurements; and (3) examine associations between DROM in the weightbearing and non-weightbearing positions and compare those between a patient group with foot and ankle injuries and a healthy group.

**Methods:**

Eighty-two patients with foot and ankle injuries (e.g., fractures, ligament and tendon injuries) and 49 healthy individuals participated in this study. Non-weightbearing DROM was measured under two different conditions: prone position with knee extended and prone position with knee flexed. Weightbearing DROM was measured as the tibia inclination angle (weightbearing angle) and distance between the big toe and wall (weightbearing distance) at maximum dorsiflexion. The effects of side (injured, uninjured) and measurement method on DROM in the patient groups were assessed using two-way repeated-measures ANOVA and *t*-tests. Pearson correlations between measurements were assessed. In addition, we analyzed whether patients without non-weightbearing DROM limitation (≤ 3 degrees) showed limitations in weightbearing DROM using *t*-tests with *Bonferroni* correction.

**Results:**

DROM in patient groups differed significantly between legs with all measurement methods (all: *P* < 0.001), with the largest effect size for weightbearing angle (*d* = 0.95). Patients without non-weightbearing DROM limitation (n = 37) displayed significantly smaller weightbearing angle and weightbearing distance on the injured side than on the uninjured side (*P* < 0.001 each), with large effect sizes (*d* = 0.97–1.06). Correlation coefficients between DROM in non-weightbearing and weightbearing positions were very weak (*R* = 0.17, *P* = 0.123) to moderate (*R* = 0.26–0.49, *P* < 0.05) for the patient group, and moderate to strong for the healthy group (*R* = 0.51–0.69, *P* < 0.05).

**Conclusions:**

DROM limitations due to foot and ankle injuries may be overlooked if measurements are only taken in the non-weightbearing position and should also be measured in the weightbearing position. Furthermore, DROM measurements in non-weightbearing and weightbearing positions may assess different characteristics, particularly in patient group.

**Level of evidence:**

Level IV, cross-sectional study.

## Introduction

Adequate ankle dorsiflexion range of motion (DROM) is necessary to perform activities of daily living and sports, such as walking, descending steps, squatting and running [1, 2]. DROM is often restricted after injuries to bones, ligaments, and tendons around the ankle and foot [3–7]. Restriction of the DROM is a predictor of future functional disability after ankle fracture [3, 4] and is also associated with talar cartilage deformity among individuals with chronic ankle instability [8]. Other previous studies have also reported that deficits in DROM increase the risks of ankle sprain and Achilles tendinopathy [9, 10]. Assessment of DROM is therefore important for foot and ankle injuries.

In clinical practice, DROM is commonly assessed in patients with foot and ankle injuries (e.g., fractures, ligament and tendon injuries). To date, DROM has been assessed using a variety of methods, including knee extension, knee flexion, non-weightbearing, and weightbearing positions [11–13]. For example, DROM limitation during knee extension can be attributed primarily to the gastrocnemius muscle [14]. If the limiting factor is something other than the gastrocnemius muscle, measurements in knee extended position may not adequately detect the limitation to DROM. In addition, DROM limitations may be more evident in the weightbearing than in the non-weightbearing position, because DROM is significantly greater in the weightbearing than in the non-weightbearing position [12]. In the weightbearing position compared to the non-weightbearing position, greater ankle moment and a greater contribution of foot motion are thought to lead to greater DROM [12]. However, for patients with foot and ankle injuries, it remains unclear which assessment methods are more likely to detect dorsiflexion limitation. If the DROM is not assessed with appropriate measurement methods, it could lead to underestimation or overlooking of DROM limitations. This will lead to inappropriate treatment programs. Identifying measurement methods that can detect greater DROM limitation in patients with foot and ankle injuries is therefore necessary.

DROM in the weightbearing position is more than twice that in the non-weightbearing position [12], and DROMs in these two positions are moderately correlated (*R* = 0.60–0.67) in healthy individuals [12, 13]. This means that while these measurements are correlated, they are not considered the same [12]. In addition, the correlation coefficient between DROMs in non-weightbearing and weightbearing positions is reported to be negligibly weak (*R* = 0.01) among individuals with diabetes [13]. Such differences in correlation characteristics from healthy individuals may be due to a stiffer tendon structure [13]. Patients with foot and ankle injuries may have stiffness, malalignment, and adhesions in the tissues around the ankle due to injuries, surgery, or joint immobilization that would limit DROM. However, the differences and relationships between DROM in non-weightbearing and weightbearing positions in patients with injuries of the foot and ankle remain unclear.

The objectives of this study were: (1) to identify assessment methods that can detect greater DROM limitation in the injured limb in patients with foot and ankle injuries; and (2) to examine associations between DROMs in weightbearing and non-weightbearing positions and compare these between a patient group with foot and ankle injuries and a healthy group. We hypothesized that: (1) DROM limitations (differences between legs) would be greater in the weightbearing position than in the non-weightbearing position; (2) even if no differences in DROM were present between the legs in the non-weightbearing position, differences would be identifiable in the weightbearing position; and (3) the correlation between DROMs in weightbearing and non-weightbearing positions would be weaker in the injured limb of the patient group than in the healthy group.

## Methods

### Participants

This cross-sectional study was conducted in a multicenter setting. A priori sample size calculations required a minimum of 34 participants in the paired *t*-test model with a moderate effect size (*d* = 0.5) using a significance level of 0.05, with statistical power of 0.8 to detect differences between injured and uninjured sides of ankle DROM. To test our second hypothesis that there would be no limitations in non-weightbearing DROM but a limitation in weightbearing DROM, we needed to identify a subgroup of patients with no difference in non-weightbearing DROM. To exceed the minimum of 34 patients required for this subgroup, we enrolled a larger number of patients. A total of 131 participants from six medical centers (i.e., hospitals and clinics), five universities, and one high school participated in this study. For the group of patients with musculoskeletal injuries of the foot and ankle (Patient group), 82 patients agreed to participate in the study. In addition, 49 healthy participants agreed to participate in the study (Healthy group). Demographic and background characteristics for all groups are shown in Table 1. All patients were diagnosed with unilateral foot and ankle injuries in clinical examinations by orthopedic surgeons in hospitals or clinics. Information on foot and ankle injuries in the Patient groups is presented in Table 1. Inclusion criteria for the Patient group were as follows: with no set weightbearing restriction, no pain during weightbearing, and the ability to walk and stand on a single leg. We included patients with any foot or ankle injury that could potentially result in DROM limitation. Healthy participants were required to have no history of foot or ankle injury and to be currently performing normal daily activities. Exclusion criteria for all participants were as follows: limited dorsiflexion due to a body part other than the ankle; difficult weightbearing; bilateral foot or ankle joint injuries; or pregnancy (due to difficulty lying prone). This study was conducted after obtaining approval from the ethics committee of Niigata University of Health and Welfare. All participants provided informed consent before participating in this study.

**Table 1:** Demographic and characteristic data

	Patients (n = 82)	Healthy (n = 49)	*P*-value
Age (years)	45.5 (19.8)	26.3 (14.2)	** < 0.001**^a^
Height (cm)	164.3 (9.2)	166.1 (7.7)	0.229^a^
Weight (kg)	63.6 (14.2)	59.6 (11.6)	0.096^a^
Sex (male/female)	40/42	21/28	0.511^b^
Surgical history (n)	40	0	
Injury type (n)			
Fractures	40	0	
Ligament injuries	12	0	
Achilles tendon injury	10	0	
Plantar fasciitis	10	0	
Osteoarthritis	3	0	
MTSS	2	0	
Hallux valgus	2	0	
Peroneal tendon injury	2	0	
PAIS	1	0	

Data represent mean (standard deviation) or number. Bold font indicates a significant difference (*P* < 0.05)

*MTSS* medial tibial stress syndrome, *PAIS* posterior ankle impingement syndrome

^a^Result from t-test

^b^Result from Pearson's chi-square test

### Procedures

The measurement methods were discussed and standardized in meetings prior to the study so that all examiners used the following methods. All measurements were taken by certified physical therapists.

**Fig. 1:**
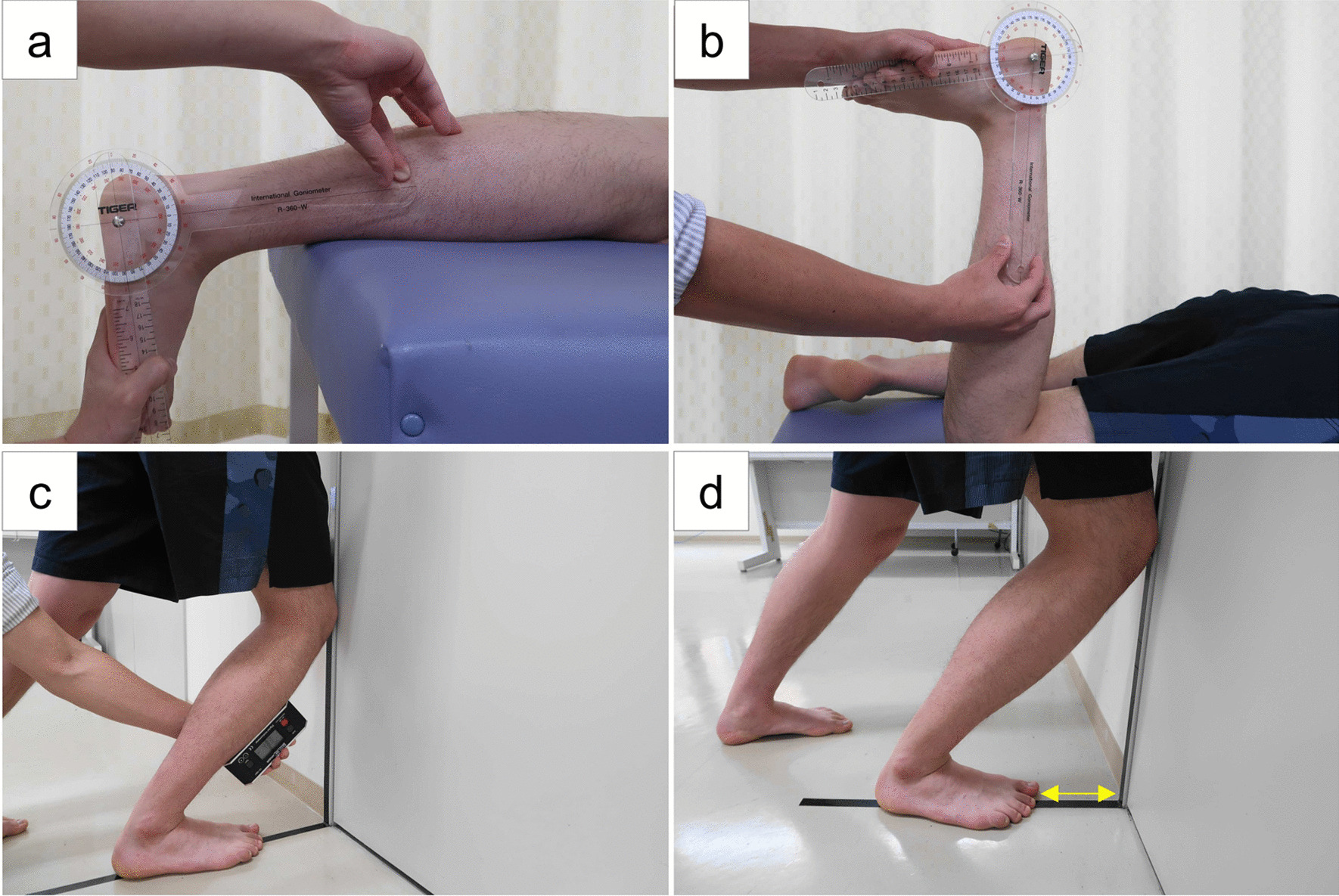
Four measurements of dorsiflexion range of motion. (**A**) Non-weightbearing with knee extended; (**B**) non-weightbearing with knee flexed 90 degrees; (**C**) weightbearing angle measurement; and (**D**) weightbearing distance measurement

DROM in the non-weightbearing position was measured in the prone position with the knee extended (NWB with knee extension) and the knee flexed 90 degrees (NWB with knee flexion), as shown in Fig. 1 [9, 12, 15, 16]. The examiner dorsiflexed the participant's ankle joint by manually applying force to the plantar metatarsal heads and measured the angle in 1-degree increments using a universal goniometer (R-360-W; Tiger Medical Instruments Co., Yao, Japan). The angle of the lateral border of the foot to the midline of the long axis of the fibula was defined as the dorsiflexion angle. Measurements were obtained in such a way that internal and external rotation, inversion and abduction of the ankle did not occur. These were visually inspected to ensure that the foot was not rotating against the lower leg and that the plantar surface was not tilted, and were manually fixed to maintain this position. One measurement of each was used for data analysis. The inter-tester reliability of these non-weightbearing DROM measurements has been reported as excellent [16–19].

The lunge test was used to measure DROM in the weightbearing position (Fig. 1). Lines on the wall and floor were marked with tape. The participant placed the foot with the second toe and center of the heel straight on the floor line. The participant then lunged forward so that the center of the patella was closer to the line of the wall. After adjusting foot position up to 5 times to obtain the maximum dorsiflexion angle at which the heel did not lift [20], the inclination angle of the lower leg was measured as the dorsiflexion angle (WB angle). This inclination angle was measured in 1-degree increments by placing a Baseline® digital inclinometer (Fabrication Enterprises Inc., White Plains, NY, USA) 15 cm below the tibial tuberosity. The distance between the big toe and wall was also measured in millimeters (WB distance). One measurement of each was used for data analysis [20]. The inter-tester reliability of these weightbearing DROM measurements has been shown to be excellent [21].

### Statistical analysis

All statistical analyses were performed using IBM SPSS Statistics 26 (IBM Corp., Armonk, NY, USA). Independent t-tests were used to compare age, height, and weight among the two groups. Chi-squared tests were used for group comparisons of sex ratios. The significance level was set at 0.05.

To test the first hypothesis, two-way repeated-measures ANOVA was used to assess the effects of measurement method (NWB with knee extension, NWB with knee flexion, and WB angle) and side (injured side, uninjured side) on dorsiflexion angles. Comparisons between measurement method or side were performed using *Bonferroni* post hoc testing when significant main effects or interactions were observed (*α* = 0.05). Since the unit of measurement differed only for WB distance (millimeters), a separate statistical analysis was performed using a paired t-test (*α* = 0.05). The effect size of the difference between legs (Cohen's *d*) was calculated and interpreted as 0.2 for small, 0.5 for medium, and 0.8 for large [22].

To test the second hypothesis, we first defined participants with a difference of ≤ 3 degrees between injured and uninjured sides in NWB with knee extension or knee flexion in the Patient group as having no limitation on non-weightbearing DROM. This threshold of 3 degrees was determined based on the standard error of measurement (1.6–2.2 degrees) and inter-examiner absolute difference (2.44 degrees) for non-weightbearing DROM measurements reported in previous studies [16, 23]. In the group with no DROM restriction in NWB with knee extension or knee flexion, measurements on the injured and uninjured sides in each measurement method (WB angle, WB distance) were compared by paired *t*-test with *Bonferroni* correction (*α* = 0.0125).

To test the last hypothesis, we assessed the association between WB angle and WB distance and the respective measures of NWB with knee extension and NWB with knee flexion using Pearson correlation analysis in two groups: the injured side of the Patient group, and the right side of the Healthy group (*α* = 0.05). Interpretations of coefficients were as follows: very strong (0.80–1.00); strong (0.60–0.79); moderate (0.40–0.59); weak (0.20–0.39); and very weak (0–0.19) [24]. Correlation coefficients were compared between the two groups using Fisher's *z* transformation. The level of significance for these comparisons was set at 0.05.

## Results

### Demographic data

Height, weight, and sex ratio did not differ significantly among the three groups (Table 1). Age was significantly higher in the Patient group than in the Healthy group (Table 1).

### Effects of measurement method and side on DROM

For the Patient group, significant main effects of measurement method (*P* < 0.001), side (*P* < 0.001), and interaction (*P* < 0.001) were found for dorsiflexion angle. For the between-side differences, post hoc analysis revealed that dorsiflexion angles differed significantly between injured and uninjured sides in NWB with knee extension, NWB with knee flexion, and WB angle (*P* < 0.001; Table 2). For differences between measurement methods, dorsiflexion angles were significantly greater for NWB with knee extension, NWB with knee flexion, and WB angle, in ascending order (*P* < 0.001). For WB distance, distances between the injured and uninjured sides also differed significantly (*P* < 0.001; Table 2). The effect size of differences in DROM measurements between injured and uninjured sides in each measurement method was largest for WB angle (*d* = 0.95).

**Table 2:** Measurement data for DROM on the injured and uninjured sides in the Patient group with each measurement method

	Injured	Uninjured	Mean difference	*P*-value	Effect size
NWB with knee extension (degrees)	9.3 (6.6)	11.7 (5.3)	2.4 (4.0)	** < 0.001**^a^	0.59
NWB with knee flexion (degrees)	17.8 (8.8)	22.2 (8.0)	4.4 (5.3)	** < 0.001**^a^	0.83
WB angle (degrees)	41.0 (8.8)	47.7 (6.3)	6.7 (7.1)	** < 0.001**^a^	0.95
WB distance (mm)	84.7 (42.5)	116.8 (30.8)	32.2 (36.8)	** < 0.001**^b^	0.87

Data represent mean (standard deviation). Bold font indicates a significant difference (*P* < 0.05)

*DROM* dorsiflexion range of motion; *NWB with knee extension* non-weightbearing position with knee extended, *NWB with knee flexion* non-weightbearing position with knee flexed, *WB angle* weightbearing position angle; *WB distance* weightbearing position distance

^a^Result from post hoc analysis

^b^Result from paired t-test

### In patients without non-weightbearing DROM limitations

In the Patient group, 48 patients showed no DROM limitations in NWB with knee extension. These patients showed significant differences in DROM measurements between the injured and uninjured sides for all NWB with knee flexion, WB angle, and WB distance (*P* < 0.001; Table 3). Effect sizes of the difference were large for WB angle and WB distance (*d* = 1.06 and 1.02). In NWB with knee flexion, 37 patients showed no dorsiflexion limitation. These patients showed significant differences in DROM measurements between injured and uninjured sides in WB angle and WB distance, and the effect sizes of these differences were large (*P* < 0.001, *d* = 0.98 and 0.97; Table 3).

**Table 3:** Measurement data in patients without DROM limitation in the non-weightbearing position

	Injured	Uninjured	Mean difference	*P*-value	Effect size
*Patients with no DROM limitation in NWB with knee extension (n* = *48)*					
NWB with knee extension (degrees)	10.6 (6.0)	11.1 (5.8)	0.6 (1.6)	0.020	0.35
NWB with knee flexion (degrees)	19.6 (9.4)	22.8 (9.2)	3.2 (4.6)	** < 0.001**	0.69
WB angle (degrees)	42.3 (7.7)	48.3 (6.8)	6.0 (5.6)	** < 0.001**	1.06
WB distance (mm)	89.1 (39.1)	120.8 (32.1)	31.7 (31.2)	** < 0.001**	1.02
*Patients with no DROM limitation in NWB with knee flexion (n* = *37)*					
NWB with knee extension (degrees)	10.4 (7.8)	11.3 (6.1)	0.9 (3.9)	0.156	0.24
NWB with knee flexion (degrees)	20.3 (9.9)	21.0 (9.9)	0.7 (2.0)	0.037	0.40
WB angle (degrees)	43.0 (7.5)	47.2 (7.0)	4.2 (4.3)	** < 0.001**	0.98
WB distance (mm)	96.1 (37.0)	115.0 (32.9)	18.9 (19.5)	** < 0.001**	0.97

Data represent mean (standard deviation). Bold font indicates a significant difference (*P* < 0.0125)

*DROM* dorsiflexion range of motion, *NWB with knee extension* non-weightbearing position with knee extended, *NWB with knee flexion* non-weightbearing position with knee flexed, *WB angle* weightbearing position angle, *WB distance* weightbearing position distance

### Correlations between DROM in weightbearing and non-weightbearing positions

In the Patient group, NWB with knee extension showed no correlation with WB angle (*R* = 0.17, *P* = 0.123) and a significant but weak correlation with WB distance (*R* = 0.26; Table 4). NWB with knee flexion correlated moderately with both WB angle and WB distance in the Patient group (*R* = 0.45 and 0.49; Table 4). The Healthy groups showed moderate to strong correlations (*R* = 0.51–0.69; Table 4). In the comparison of correlation coefficients, the correlation of dorsiflexion angles in NWB with knee extension and WB angle was significantly smaller in the Patient group than in the Healthy group (*P* = 0.013, Table 4).

**Table 4:** Correlation coefficients between DROM measurements in the non-weightbearing and weightbearing positions

	*R*-value		*P*-value^a^
	Patient	Healthy	Patient vs. Healthy
*Correlations with NWB with knee extension*			
WB angle	0.17	0.56*	**0.013**
WB distance	0.26*	0.51*	0.110
*Correlations with NWB with knee flexion*			
WB angle	0.45*	0.58*	0.338
WB distance	0.49*	0.69*	0.093

*DROM* dorsiflexion range of motion, *NWB with knee extension* non-weightbearing position with knee extended, *NWB with knee flexion* non-weightbearing position with knee flexed; *WB angle* weightbearing position angle; *WB distance* weightbearing position distance

^*^Indicates a significant correlation (*P* < 0.05)

^a^Comparison results using Fisher's z-transform. Bold font indicates a significant difference (*P* < 0.05)

## Discussion

The main findings of this study were: (1) differences in DROM between injured and uninjured sides were significant for all measures, and the effect size was greater in the weightbearing position in the Patient group; (2) even in patients with no difference in DROM between injured and uninjured sides in the non-weightbearing position, the difference was significant and large in the weightbearing position; and (3) correlations between measurements in the non-weightbearing and weightbearing positions tended to be weak in the Patient group, unlike in the Healthy group.

The results of each measurement method suggest that a large difference in DROM between the legs can be detected in NWB with knee flexion, WB angle, and WB distance in the Patient group (Table 2). These results partially supported our hypothesis 1. In the Patient group, the difference between legs was increased by knee flexion in the non-weightbearing position, suggesting that factors other than the gastrocnemius muscle may be more involved in DROM limitations. In addition, in the weightbearing position, greater torque is applied to the ankle [12], and the effects of other joint motions (e.g., subtalar and midtarsal joints) and muscle activity due to loading may contribute to greater limitations on DROM. Measurement in the weightbearing position would be recommended because DROM limitations may be overlooked when measurements are obtained only in the non-weightbearing position.

Even among patients with no DROM limitations in the non-weightbearing position, limitations were observed in the weightbearing position. The weightbearing DROM of these patients was similar to that of the ankle injury group in the previous studies [25, 26]. This finding supported our hypothesis 2. This finding suggests that assessing DROM only in the non-weightbearing position is inadequate and that assessment of DROM in the weightbearing position is necessary. This finding also suggests that DROM limitations may have improved in the non-weightbearing position, but not yet in the weightbearing position. Intervention programs following foot and ankle injuries would need to be designed while keeping in mind the possibility of residual DROM limitations in the weightbearing position.

The correlation between measurements in the non-weightbearing and weightbearing positions tended to be weak in the Patient group, unlike in the Healthy group. These results supported our hypothesis 3. The correlation coefficient between NWB with knee extension and weightbearing position was particularly weak, and that in the Patient group was significantly smaller than that in the Healthy group. Correlations for the Healthy group were moderate to strong, as in previous studies (*R* = 0.60–0.67) [12, 13], suggesting that DROM in the non-weightbearing and weightbearing positions assesses different phenomena [12]. Our findings suggest that foot and ankle injuries further confound the association between DROM in the non-weightbearing and weightbearing positions. This may be because injuries result in different factors limiting dorsiflexion than those seen in healthy individuals. The results suggest that DROM assessment differs between non-weightbearing and weightbearing positions, particularly in those with foot and ankle injury.

Regarding clinical relevance, DROM should be measured in non-weightbearing and weightbearing positions in patients with foot and ankle injuries, because these measurements do not correlate and may assess different DROM limiting factors. It should also be noted that measuring only at the non-weightbearing position is not sufficient. This is because even if the DROM is not restricted in the non-weightbearing position, it may be restricted in the weightbearing position. In addition, clinicians may need to intervene to account for the possibility of more residual DROM limitations in the weightbearing position in patients with foot and ankle injuries.

This study has several limitations. First, when weightbearing positions were measured, load amounts were not standardized. Differences in the amount of load could have affected DROM. Second, the types of foot and ankle injuries varied widely in this study. Different types of conditions may have different DROM characteristics. Finally, the Patient group was older than the healthy group. Age may thus have affected tendon stiffness during dorsiflexion.

## Conclusions

The difference in DROM between injured and uninjured sides for the Patient group was significant in both weightbearing and non-weightbearing positions, and the difference was greater in the weightbearing position. Patients with no difference in DROM between the legs in the non-weightbearing position were also found to show significant and large differences in DROM in the weightbearing position. In addition, the correlation between DROMs in the non-weightbearing and weightbearing positions tended to be weaker in the Patient group, unlike in the Healthy group.

## Data Availability

The datasets used and/or analyzed during the current study are available from the corresponding author on reasonable request.
